# Psychometric properties and age- and gender-specific normative values of the Patient Health Questionnaire-4 among older adults aged 60 years and above

**DOI:** 10.1186/s12877-026-08172-5

**Published:** 2026-08-28

**Authors:** Nikolay Dimitrov, Elmar Brähler, Andreas Hinz, Bjarne Schmalbach, Jörg M. Fegert, Markus Zenger

**Affiliations:** 1 Faculty of Applied Human Studies, University of Applied Sciences Magdeburg-Stendal, Stendal, Germany; 2 Clinic for Psychosomatic Medicine and Psychotherapy, University Medical Center Johannes Gutenberg University Mainz, Mainz, D-55131 Germany; 3 Clinic and Polyclinic for Psychosomatic Medicine and Psychotherapy, Rostock University Medical Center, Rostock, D-18147 Germany; 4 Department of Medical Psychology and Medical Sociology, University of Leipzig, Leipzig, Germany; 5 Medical Psychology & Medical Sociology, University Medical Center of the Johannes Gutenberg-University Mainz, Mainz, Germany; 6 Department of Child and Adolescent Psychiatry, Psychosomatics and Psychotherapy, University Hospital of Ulm, Ulm, Germany; 7 German Center for Mental Health (DZPG), partner site Mannheim-Heidelberg-Ulm, Mannheim, Germany; 8 Integrated Research and Treatment Center Adiposity Diseases - Behavioral Medicine, Psychosomatic Medicine and Psychotherapy, University of Leipzig Medical Center, Leipzig, Germany

**Keywords:** PHQ-4, PHQ-2, GAD-2, Older adults, Normative values, Psychometric properties, Depression, Anxiety

## Abstract

**Background:**

Mental health problems in older adults are often underrecognized despite their substantial impact on health and well-being. Brief, valid screening tools with age-specific normative data are needed for this growing population. This study examined the psychometric properties of the Patient Health Questionnaire-4 (PHQ-4) and established age- and gender-specific normative values for adults aged 60 years and older in Germany.

**Methods:**

Data were drawn from four nationally representative face-to-face household surveys conducted in Germany between 2021 and 2024. Four of the total 3,212 participants were excluded due to missing PHQ-4 responses, resulting in a final sample of *N* = 3,208. The psychometric properties of the Generalized Anxiety Disorder-2 (GAD-2), the Patient Health Questionnaire-2 (PHQ-2), and the Patient Health Questionnaire-4 (PHQ-4) were evaluated.

**Results:**

The PHQ-4 showed excellent internal consistency. The correlated two-factor model, distinguishing depression and anxiety symptoms, demonstrated excellent fit. Measurement invariance was supported across gender and age groups. Women reported higher overall psychological distress than men, mainly due to higher anxiety symptoms, and PHQ-4 scores were highest in the oldest age group. Convergent validity was supported by moderate to strong negative correlations with quality of life, resilience, sense of coherence, and well-being. Age- and gender-specific normative values were provided for the PHQ-2, GAD-2, and PHQ-4.

**Conclusions:**

The PHQ-4 is a reliable and valid ultra-brief screening instrument for psychological distress. The age- and gender-specific normative values provided here may improve score interpretation for community-dwelling older adults in Germany and help identify individuals who may require further clinical assessment.

**Supplementary Information:**

The online version contains supplementary material available at https://doi.org/10.1186/s12877-026-08172-5.

## Background

Mental health disorders in older adults are frequently overlooked and inadequately treated, with stigma—alongside other barriers—often discouraging individuals from seeking the professional support they need [1]. One of the main challenges in providing appropriate mental health care to older adults is distinguishing between adaptive and pathological symptoms, which are frequently misinterpreted as normal, short-term responses to the aging process [2, 3].

Given the growing number of older adults and the unique challenges associated with population aging [4–6], the development of age-specific normative data for mental health screening assessments is an essential first step toward improving mental health care for older adults. The current study responds to this need by examining the psychometric properties of the Patient Health Questionnaire-4 (PHQ-4), an ultra-brief screening instrument for psychological distress caused by symptoms of anxiety and depression, and by providing gender- and age-specific normative values for the German population aged 60 and over.

### Depression and anxiety in older adults

Depression and anxiety are among the most commonly experienced mental health issues in later life, yet they often go undetected [7–9]. Globally, the prevalence of depression was estimated at around 19.2% and anxiety at around 16.5% in the elderly population [10]. Depression and anxiety can have a significant negative impact on the well-being [11], health impairment [12], and mortality of older adults [13]. Comorbidity between anxiety and depression is common in later life [14]. When depression and anxiety occur together, their combined impact on everyday life is even more severe than the sum of their individual effects [15]. Because the symptoms of depression and anxiety experienced by older adults are often age-specific, these mental health disorders go undiagnosed and untreated in old age, despite their impact on mental and physical health [7, 16].

### Risk factors for depression and anxiety in older adults

The development of depression and anxiety in older adults is commonly conceptualized within a biopsychosocial framework, in which biological, psychological, and social vulnerabilities interact [17, 18]. Biologically, age-related alterations in brain networks and structure, cerebrovascular disease, and immune-inflammatory dysregulation have been implicated particularly in late-life depression [19]. Psychologically, helplessness-related cognitive patterns, together with the cumulative impact of bereavement, disability, functional decline, and other losses, may contribute to depressive and anxiety symptoms [20, 21]. Socially, limited social support, loneliness, and role transitions associated with widowhood, involuntary retirement, or declining health can further increase vulnerability [21–23]. These mechanisms operate dynamically rather than independently, helping to explain the substantial overlap between risk factors for late-life depression and anxiety [17, 18].

Risk factors for depression have been more extensively researched than those for anxiety [18]. Older women seem to be at greater risk for both depression and anxiety [24]. Chronic physical illnesses are associated with both mental disorders in later life [25, 26]. Moreover, stressful life events can often lead to the onset of anxiety and depression in older adults [18, 27].

Lower levels of education have been associated with a higher risk of both conditions [25]. Additionally, adverse childhood experiences, such as trauma or neglect, have been found to increase the likelihood of developing anxiety and depression disorders [28].

Physical limitations, such as difficulties with mobility, are linked to both anxiety and depression [29, 30]. Neuroticism has been identified as a predictor for depression as well as for depression and anxiety occurring together [18].

There are also some differences found between the risk factors for depression and anxiety in old age. Low contact frequency, childlessness, and lower income were linked only to anxiety, while a smaller social network and being unmarried were associated with depression, but not anxiety [18].

### Outcomes of depression and anxiety in older adults

Depression and anxiety in older adults are associated with a wide range of serious negative outcomes affecting mental health, physical health, cognitive function, and overall well-being. They can also lead to substantial costs for the healthcare system.

Depression and anxiety are both linked to suicidal thoughts and suicide attempts among older adults [31]. In older adults, depression is associated with negative subjective health [32] and with a higher likelihood of disability [33], increased mortality rates [34], and greater susceptibility to frailty [35]. It can also accelerate cognitive decline [36] and the development of Alzheimer’s disease [37].

Anxiety causes considerable subjective distress [38], leads to reduced life satisfaction and increases the likelihood of disability [39]. Additionally, anxiety raises the risk of mortality, particularly from cardiovascular diseases [40]. Population-based studies have shown that depression and anxiety in older adults are associated with substantially increased healthcare utilization and costs, placing a significant burden on healthcare systems [41].

### Screening measures

The initial step for providing adequate treatment for mental health disorders in older age is effective screening. This highlights the need for well-validated screening tools with detailed normative values for older adults, as they are particularly vulnerable to depression and anxiety, due to the more severe outcomes associated with their age group. Individuals who screen positive should subsequently be evaluated using more detailed, clinically validated diagnostic instruments to confirm the presence and severity of mental health disorders and to guide appropriate intervention.

Although there are well-validated, longer instruments for assessing depression and anxiety (e.g. the Beck Depression Inventory–II [42] and the State–Trait Anxiety Inventory [43]), current trends are shifting toward ultra-brief screening tools due to their numerous advantages - like time efficiency and low respondent burden - while minimally compromising psychometric properties [44].

PHQ-4 is a brief and efficient screening tool designed to assess psychological distress through symptoms of depression and anxiety, developed by Kroenke, Spitzer, Williams, and Löwe in 2009 [45]. It comprises just four items, divided into two subscales: two items assess depression (Patient Health Questionnaire-2, PHQ-2) and two assess anxiety (Generalized Anxiety Disorder-2, GAD-2).

The PHQ-2 is a well-validated ultra-brief screening tool derived from the PHQ-9, focusing specifically on the two main symptoms of depression: depressed mood and loss of interest [46]. Similarly, GAD-2 was introduced as a short version of the Generalized Anxiety Disorder-7. The two items assess the two core symptoms of generalized anxiety: persistent nervousness and uncontrollable worry [47]. It has been validated in various contexts and has demonstrated good psychometric properties in screening for generalized anxiety disorder, panic disorder, social anxiety disorder, and posttraumatic stress disorder [47–50].

PHQ-4 enables healthcare providers to quickly determine the need for further diagnostic evaluation, facilitating early detection and treatment of potential mental health problems. Normative data for the PHQ-4, based on a representative sample from the general population, were first published by Löwe et al. in 2010 [51] and updated in 2022 [52]. To the best of our knowledge, PHQ-4 has not yet been validated in a sample of older adults, and no detailed normative data for this age group have been published.

The current study aims to evaluate the psychometric properties and provide age- and gender-specific normative values for GAD-2, PHQ-2 and PHQ-4 for German older adults.

## Methods

### Participants and samples analyzed

The present study is based on four nationally representative, face-to-face household surveys conducted in Germany between 2021 and 2024. The data collection was conducted by the Independent Service for Surveys, Methods and Analyses (USUMA, Berlin).

A multi-stage sampling procedure ensured that the samples were representative of the general population in Germany. In the first stage, geographically stratified sampling points were selected across the country. In the second stage, households were chosen within each sampling point using a random-route technique. In the final stage, one eligible respondent was randomly selected from each household to participate. Each address was contacted up to two times before being classified as unreachable.

Only data from participants aged 60 years or older at the time of data collection were analyzed. Participants were personally asked about demographic characteristics, followed by self-report questionnaires, which included the German version of the PHQ-4. Gender was assessed with a single item asking participants to indicate their gender, with the response categories “male”, “female”, and “diverse”.

The fieldwork response rates for the complete source surveys were 42.6% in 2021, 41.2% in 2022, 41.3% in 2023, and 39.4% in 2023–2024. Because age-specific data for nonresponders were unavailable, response rates specifically for adults aged 60 years and older could not be calculated, and the extent of potential nonresponse and selection bias in this age group could not be determined.

All participants were informed about the purpose and procedures of the study and provided written informed consent prior to participation. The surveys were approved by the Ethics Committee of the Medical Faculty of the University of Leipzig with the following reference numbers: 298/21-ek for 2021, 594/21-ek for 2022, 355/22-ek for 2023, and 278/23-ek for 2024.

### Instrument

The PHQ-4 consists of four items that assess the frequency of core anxiety and depression symptoms experienced over the past two weeks. It has two subscales: the PHQ-2 (Items 1 and 2, depressive symptoms) and the GAD-2 (Items 3 and 4, anxiety symptoms). Participants indicated how often they experienced each symptom in the past two weeks on a 4-point Likert scale: 0 (Not at all), 1 (Several days), 2 (More than half the days), 3 (Nearly every day). The four items are: (1) Little interest or pleasure in doing things, (2) Feeling down, depressed, or hopeless, (3) Feeling nervous, anxious, or on edge, and (4) Not being able to stop or control worrying.

The PHQ-4 total score ranges from 0 to 12, with higher scores indicating greater psychological distress. Additionally, subscale scores for PHQ-2 (range 0–6) and GAD-2 (range 0–6) can be calculated. The total and subscale scores were used to derive normative values for different age and gender groups and to examine group differences. The standard method of obtaining percentile ranks was applied [53]. Normative values were computed using unweighted data.

### Brief description of the instruments used to test the convergent validity

To examine convergent validity, correlations between PHQ-4 scores and various other instruments were analyzed. The data used in the present study were derived from four separate data collections, each employing different sets of measures. As a result, the PHQ-4 was compared with different scales across datasets, depending on the available instruments.

#### European health interview survey-quality of life 8-item index

The European Health Interview Survey-Quality of Life 8-item index (EUROHIS-QOL [54–56]) is a brief measure of subjective quality of life. It consists of eight items covering physical, psychological, social, and environmental domains. Responses are given on a 5-point Likert scale ranging from 1 (“Very poor”/“Not at all”) to 5 (“Very good”/“Completely”), depending on the item. A total mean score is calculated, with higher scores indicating better perceived quality of life. In the present sample, Cronbach’s α was 0.90.

#### Brief resilience scale

The Brief Resilience Scale (BRS [57, 58]) is a six-item scale that assesses the ability to recover from stress and adversity. The scale includes three positive and three negatively worded items. Responses were given on a 5-point Likert scale ranging from 1 (“Strongly disagree”) to 5 (“Strongly agree”). A mean score is calculated, with higher scores indicating greater resilience. In the present sample, Cronbach’s α was 0.87.

#### Sense of coherence scale–9

The Sense of Coherence Scale–9 (SOC-L9 [59]) assesses a sense of coherence across three dimensions: comprehensibility, manageability, and meaningfulness. It includes nine items rated on 7-point scales. A mean score is calculated, with higher scores indicating stronger sense of coherence. In the present sample, Cronbach’s α was 0.90.

#### WHO-5 well-being index

The WHO-5 Well-Being Index (WHO-5 [54, 60, 61]) consists of five items assessing positive emotional well-being over the past two weeks, such as feeling cheerful, calm and relaxed, active and vigorous, rested upon waking, and that life is filled with interest. Responses range from 0 (“At no time”) to 5 (“All of the time”), with a sum score ranging from 0 to 25; higher scores indicate better well-being. In the present sample, Cronbach’s α was 0.95.

### Data analysis

To examine differences in psychological distress across demographic groups, an independent-samples t-test was performed to assess differences by gender, and separate one-way analyses of variance (ANOVAs) were conducted to examine differences by age group and survey wave. Welch’s t-test was used when the homogeneity-of-variance assumption was violated. These analyses were performed for the PHQ-4 total score and separately for the PHQ-2 and GAD-2 subscales. The two participants who selected the diverse response category were excluded only from gender-group comparisons because the group was too small for meaningful comparison. They were retained in overall, age-group, survey-wave, psychometric, and normative analyses. Furthermore, the internal consistency of the PHQ-4 and its subscales was assessed using Cronbach’s alpha and McDonald’s omega to estimate construct reliability.

### Factor analysis

Exploratory factor analysis (EFA) was not conducted, as the factorial structure of the PHQ-4 has been extensively investigated in previous research and consistently shown to support either a one- or two-factor solution [62]. Instead, confirmatory factor analysis (CFA) was conducted to evaluate the model fit of two theoretically and empirically supported structures: (1) a unidimensional model in which all four items load onto a single general distress factor, and (2) a correlated two-factor model differentiating between anxiety and depression symptomatology.

All models were examined using the maximum likelihood method and based on their covariance matrices. Maximum likelihood estimation was chosen given the large sample size; simulation evidence indicates that ML performs comparably to categorical estimators for factor correlations and model fit even with as few as four response categories, though categorical methods may better recover individual factor loadings under these conditions [63]. To evaluate the robustness of this estimation approach, an ordinal CFA using a WLSMV estimator was additionally conducted as a sensitivity analysis. Model fit was evaluated using several indices: the chi-square statistic divided by its degrees of freedom (CMIN/DF), the comparative fit index (CFI), the Tucker–Lewis Index (TLI), the root mean square error of approximation (RMSEA), the standardized root mean square residual (SRMR), and the Bayesian Information Criterion (BIC). Acceptable model fit was defined by the following thresholds: CFI and TLI ≥ 0.95 (preferably ≥ 0.97), RMSEA ≤ 0.08, SRMR ≤ 0.05, and lower BIC values indicating better model fit [64].

To assess the robustness of the factorial structure across groups, multigroup CFA was conducted to test measurement invariance across gender and age groups. Invariance testing followed a three-step hierarchical procedure: configural invariance (no constraints), metric invariance (equality of factor loadings), and scalar invariance (equality of both factor loadings and item intercepts). Given the sensitivity of the chi-square statistic to sample size, model comparisons were based primarily on changes in CFI, with ΔCFI < 0.01 indicating acceptable invariance. Changes in RMSEA were considered supplementary because RMSEA estimates may be unstable in models with very low degrees of freedom [65]. Because the χ² statistic is highly sensitive to large sample sizes and trivial deviations from exact model fit, interpretation of invariance was based primarily on changes in approximate fit indices rather than χ² alone [66]. Latent mean differences in depression and anxiety across gender were examined using multigroup confirmatory factor analysis.

### Convergent validity

To examine the convergent validity of the PHQ-4, correlations with various other self-report instruments were analyzed. The data used in the present study were derived from four independent samples, each employing a distinct set of questionnaires. As a result, the correlations of the PHQ-4 were examined with different instruments across samples, depending on the measures available in each dataset. Only participants who provided valid responses to all items from both instruments were analyzed.

In the first dataset, the PHQ-4 was compared with the EUROHIS-QOL, which assesses subjective quality of life. Based on previous research showing that higher levels of psychological distress are associated with lower quality of life, moderate to strong negative correlations with the EUROHIS-QOL were expected [67].

In the second dataset, correlations were calculated between PHQ-4 scores and the BRS. A low to moderate negative correlation was anticipated, as higher psychological distress is usually associated with lower resilience [68].

In the third dataset, the PHQ-4 was correlated with the SOC-L9. Moderate to strong negative correlations were anticipated with SOC-L9, as greater sense of coherence is typically a protective factor for psychological distress [69].

In the fourth dataset, PHQ-4 scores were compared with the *WHO-5 Well-Being Index*. Moderate to strong negative correlation was expected, consistent with the notion that lower well-being is associated with higher anxiety and depressive symptoms [67].

All primary statistical analyses were conducted using SPSS 29 and AMOS 29. The ordinal CFA sensitivity analysis was conducted in R, using the lavaan package [70]. An α-level of 5% was applied.

## Results

### Sample characteristics

The combined sample included 3,212 participants from four representative samples collected in 2021, 2022, 2023 and 2023–2024. The 2023 and 2023–2024 waves reflect two distinct, non-overlapping fielding periods rather than a labeling inconsistency; no participant is included in more than one wave. Four participants were removed from further analysis due to not providing a valid response to at least one of the PHQ-4 items. A total of *N* = 3208 participants (1662 women, 2 diverse) were included in the main analysis. Their age at the time of data acquisition was between 60 and 101 years, with an average age of 70.04 years (SD = 7.44). Detailed demographic characteristics are provided in Table 1.

**Table 1:** Sample characteristics

		Total (*N* = 3208)	Men (*n* = 1544)	Women (*n* = 1662)
Age (years)	Mean (SD)	70 (7.44)	69.9 (7.4)	70.2 (7.5)
	Minimum	60	60	60
	Maximum	101	101	96
Year of data collection	2021	825 (25.7%)	385 (24.9%)	439 (26.4%)
	2022	768 (23.9%)	386 (25.0%)	382 (23.0%)
	2023	815 (25.4%)	378 (24.5%)	437 (26.3%)
	2023–2024	800 (24.9%)	395 (25.6%)	404 (24.3%)
Age group (years)	60–64	912 (28.4%)	453 (29.3%)	459 (27.6%)
	65–69	792 (24.7%)	376 (24.4%)	416 (25.0%)
	70–74	669 (20.9%)	341 (22.1%)	327 (19.7%)
	75–79	387 (12.1%)	165 (10.7%)	222 (13.4%)
	80+	448 (14.0%)	209 (13.5%)	238 (14.3%)
Living with a partner	Yes	1650 (51.4%)	994 (64.4%)	656 (39.5%)
	No	1516 (47.3%)	535 (34.7%)	979 (58.9%)
Years education	≤ 8 years	1423 (44.4%)	639 (41.4%)	783 (47.1%)
	9–11 years	1216 (37.9%)	570 (36.9%)	645 (38.8%)
	≥ 12 years	565 (17.6%)	334 (21.6%)	231 (13.9%)
Net equivalent household income, per month, €	< 1250	680 (21.2%)	277 (17.9%)	402 (24.2%)
	1251–1500	850 (26.5%)	409 (26.5%)	441 (26.5%)
	1501–1750	448 (14.0%)	152 (9.8%)	296 (17.8%)
	1751–2000	446 (13.9%)	257 (16.6%)	189 (11.4%)
	2001–2250	220 (6.9%)	101 (6.5%)	119 (7.2%)
	> 2251	523 (16.3%)	331 (21.4%)	191 (11.5%)
Marital Status	Married (living together)	1435 (44.7%)	877 (56.8%)	558 (33.6%)
	Married (separated)	55 (1.7%)	36 (2.3%)	19 (1.1%)
	Single	233 (7.3%)	129 (8.4%)	104 (6.3%)
	Divorced	605 (18.9%)	252 (16.3%)	352 (21.2%)
	Widowed	878 (27.4%)	249 (16.1%)	628 (37.8%)

Totals include *n* = 2 participants who selected the diverse response category, who are not shown as a separate column due to insufficient sample size for meaningful comparison; this accounts for the apparent discrepancy between the Men + Women subtotals and the Total column in some rows (e.g., age 70–74 and 80+, survey years 2021 and 2023–2024). Net equivalent household income was computed using a square-root equivalence scale based on household size

### Distribution of the PHQ-4 scores

The diverse group was too small for gender-group comparisons. Analyses by gender were therefore conducted for men and women only, whereas all other analyses retained the full sample of *N* = 3,208. Men and women differed significantly in several domains. More women than men reported not living with a partner (χ² = 258.81, *p* < .001). Women also had fewer years of schooling (χ² = 26.77, *p* < .001) and a lower equivalent household income (χ² = 61.30, *p* < .001).

The mean score on the PHQ-4 was 1.95 with a standard deviation (SD) of 2.31. Scores ranged from 0 to 12, the full range of the scale. The total PHQ-4 scores are usually classified into normal (0–2), mild (3–5), moderate (6–8), and severe (9–12). The full distribution of the scores is presented in Table 2.

**Table 2:** Distribution of the PHQ-4 scores into severity categories

PHQ-4 score	*n*	%
Normal (0–2)	2173	67.7
Mild (3–5)	773	24.1
Moderate (6–8)	198	6.2
Severe (9–12)	64	2.0

The scores on the subscales PHQ-2 and GAD-2 can also be individually interpreted. The recommended cut-off score of ≥ 3 on the PHQ-2 has been shown to identify major depressive disorder with sufficient diagnostic accuracy, yielding a sensitivity of 83% and a specificity of 90% in validation studies [46]. Similarly, for the GAD-2, a threshold of ≥ 3 is commonly applied, providing a sensitivity of 86% and a specificity of 83% for generalized anxiety disorder [47].

When applying these established subscale cut-offs to the current sample, 11.2% of participants screened positive for probable depression, whereas 8.6% screened positive for probable anxiety (Table 3). It should be noted that these figures reflect screening-based classifications derived from validated cut-off scores rather than clinical diagnoses. They indicate a probable need for further clinical evaluation rather than diagnostic prevalence.

**Table 3:** Frequency distribution of PHQ-2 and GAD-2 screening classifications

Screening classification	*n*	%
Below PHQ-2 cut-off (< 3)	2850	88.8
Positive PHQ-2 screen (≥ 3)	358	11.2
Below GAD-2 cut-off (< 3)	2932	91.4
Positive GAD-2 screen (≥ 3)	276	8.6

### Group differences

An independent-samples *t*-test revealed a significant gender difference in overall psychological distress, *t* (3204) = − 3.79, *p* < .001, *d* = 0.13, with women reporting higher PHQ-4 total scores (M = 2.10, SD = 2.32) than men (M = 1.79, SD = 2.28; see Table 4).

**Table 4:** Gender differences in PHQ-4 scores (independent samples t-test)

Measure	Men M (SD)	Women M (SD)	*p*	d
GAD-2	0.74 (1.13)	0.98 (1.21)	< 0.001	0.20
PHQ-2	1.05 (1.31)	1.12 (1.29)	0.131	0.05
PHQ-4	1.79 (2.28)	2.10 (2.32)	< 0.001	0.13

Values represent means (M) and standard deviations (SD) of the total scores. p-values are based on two-tailed independent-samples t-tests; Welch’s test was used for GAD-2 because the homogeneity-of-variance assumption was not met. Effect sizes are reported as absolute Cohen’s d values; positive values indicate higher mean scores among women

Analysis of the subscales indicated that this effect was primarily attributable to gender differences in anxiety symptoms. Women scored significantly higher on the GAD-2, Welch’s *t* (3203.97) = − 5.77, *p* < .001, *d* = 0.20. In contrast, no significant gender difference was found for depression symptoms on the PHQ-2, *t* (3204) = − 1.51, *p* = .131, *d* = 0.05.

A significant main effect of age group emerged for PHQ-4 total scores, F (4, 3203) = 14.95, *p* < .001, η² = 0.018. Mean scores were 1.77 (SD = 2.27) in the 60–64 group and highest in the 80 + group (M = 2.67, SD = 2.63; Table 5). This pattern was present in both subscales. Depressive symptoms differed significantly across age groups, F (4, 3203) = 19.42, *p* < .001, η² = 0.024. Anxiety symptoms also differed across age groups, although with a smaller effect size, F (4, 3203) = 7.59, *p* < .001, η² = 0.009.

**Table 5:** Age differences in PHQ-4 and its subscales (ANOVA)

Scale		60–64	65–69	70–74	75–79	80+	*p* (F)	η²
PHQ-2	M (SD)	0.96 (1.25)	0.93 (1.25)	1.09 (1.23)	1.19 (1.29)	1.53 (1.47)	< 0.001 (19.42)	0.024
GAD-2	M (SD)	0.80 (1.18)	0.80 (1.15)	0.82 (1.10)	0.91 (1.17)	1.14 (1.32)	< 0.001 (7.59)	0.009
PHQ-4	M (SD)	1.77 (2.27)	1.72 (2.22)	1.91 (2.14)	2.10 (2.27)	2.67 (2.63)	< 0.001 (14.95)	0.018

Values represent *M* means and *SD* standard deviations of the total scores. *p* values were calculated from one-way ANOVAs. Effect sizes are reported as eta squared (η²)

Significant differences across survey waves (2021, 2022, 2023, and 2023–2024) were observed for PHQ-4, F (3, 3204) = 10.69, *p* < .001, η² = 0.010; PHQ-2, F (3, 3204) = 9.63, *p* < .001, η² = 0.009; and GAD-2, F (3, 3204) = 10.04, *p* < .001, η² = 0.009.

### Item characteristics

Item and subscale characteristics are presented in Table 6.

**Table 6:** Item and subscale characteristics

Item	M	SD	g₁	g₂	Item–Total *r*	α if deleted	ω if deleted	α	ω
Little interest or pleasure in doing things	0.61	0.71	1.02	0.77	0.71	0.85	0.85		
Feeling down, depressed, or hopeless	0.48	0.70	1.43	1.69	0.79	0.82	0.82		
Feeling nervous, anxious, or on edge	0.46	0.65	1.30	1.39	0.69	0.86	0.86		
Not being able to stop or control worrying	0.40	0.65	1.62	2.34	0.74	0.84	0.84		
GAD-2	0.86	1.18	1.51	2.27				0.80	
PHQ-2	1.09	1.30	1.28	1.46				0.83	
PHQ-4	1.95	2.31	1.44	2.08				0.88	0.88

*M* mean, *SD* standard deviation, *g*₁ skewness, *g*₂ kurtosis, *Item–Total r* corrected item–total correlation, *α* Cronbach’s alpha, *ω* McDonald’s omega. McDonald’s omega was not calculated for the PHQ-2 and GAD-2 subscales because each consists of only two items

### Confirmatory factor analysis

Two alternative factor structures of the PHQ-4 were examined using confirmatory factor analysis. The one-factor model, in which all four items loaded on a single latent construct, yielded strong standardized loadings (0.75–0.87), but did not demonstrate acceptable overall fit according to the predefined criteria (Table 7).

**Table 7:** Fit indices for maximum-likelihood CFA models of the PHQ-4

Model	χ² (df)	CMIN/DF	CFI	SRMR	RMSEA [90%CI]	TLI	BIC
One-factor	161.68 (2)	80.84	0.976	0.027	0.158 [0.138, 0.179]	0.928	226.27
Two-factor	1.14 (1)	1.14	1.000	0.002	0.007 [0.000, 0.048]	1.000	73.80

*χ*^2^ chi-square test statistic, *df*  degrees of freedom, *CMIN/DF* chi-square divided by degrees of freedom, *CFI* comparative fit index, *SRMR* standardized root mean square residual, *RMSEA* root mean square error of approximation, *CI* confidence interval, *TLI* Tucker–Lewis index, *BIC* Bayesian information criterion

The table presents the primary maximum-likelihood analyses; results of the ordinal WLSMV sensitivity analysis are reported in the accompanying text

A two-factor model was then specified, with items 1 and 2 loading on a depression factor and items 3 and 4 loading on an anxiety factor, allowing the latent variables to correlate. This model showed a substantially improved and overall excellent fit to the data (Table 7). All items loaded strongly on their intended factors (depression: 0.79–0.90; anxiety: 0.78–0.85). The latent factors were highly correlated (*r* = .89), indicating substantial overlap while remaining empirically distinguishable.

As a sensitivity analysis, the correlated two-factor model was re-estimated using WLSMV while treating the four items as ordered-categorical indicators. The approximate fit indices indicated good model fit, scaled χ² (1) = 6.22, *p* = .013, CFI = 1.000, TLI = 0.999, RMSEA = 0.040, and SRMR = 0.005. Standardized loadings were 0.87 and 0.96 for the depression items and 0.86 and 0.93 for the anxiety items. The latent correlation between depression and anxiety was *r* = .91, closely corresponding to the maximum-likelihood estimate of *r* = .89. Thus, the substantive conclusion regarding the correlated two-factor structure was unchanged by the choice of estimator.

### Measurement invariance across gender and age

Measurement invariance of the two-factor PHQ model was examined across gender and age groups using multigroup confirmatory factor analysis (Table 8).

**Table 8:** Tests for invariance across gender and age groups

Group / model	*N*	χ² (df)	Δχ²	Δp	CMIN/DF	CFI	ΔCFI	RMSEA	ΔRMSEA
Gender									
Men	1544	5.064 (1)	—	—	5.064	0.999	—	0.051	—
Women	1662	0.761 (1)	—	—	0.761	1.000	—	< 0.001	—
Model									
Configural	—	5.826 (2)	—	—	2.913	0.999	—	0.024	—
Metric	—	7.050 (4)	1.224	0.542	1.762	1.000	0.001	0.015	0.009
Scalar	—	18.419 (7)	11.369	0.010	2.631	0.998	0.002	0.023	0.008
Age									
60–64 years	912	0.687 (1)	—	—	0.687	1.000	—	0.000	—
65–69 years	792	0.698 (1)	—	—	0.698	1.000	—	0.000	—
70–74 years	669	6.170 (1)	—	0.013	6.170	0.996	—	0.088	—
75–79 years	387	0.042 (1)	—	—	0.042	1.000	—	0.000	—
80 + years	448	0.015 (1)	—	—	0.015	1.000	—	0.000	—
Model									
Configural	—	7.612 (5)	—	—	1.522	1.000	—	0.013	—
Metric	—	13.136 (13)	5.524	0.700	1.010	1.000	< 0.001	0.002	0.011
Scalar	—	45.326 (25)	32.190	0.001	1.813	0.997	0.003	0.016	0.014

*χ*^2^ chi-square test statistic, *df* degrees of freedom, Δ*χ*^2^ chi-square difference between nested models, Δ*p* *p* value associated with the chi-square difference test, *CMIN/DF* chi-square divided by degrees of freedom, *CFI* comparative fit index, Δ*CFI* change in CFI relative to the less constrained model, *RMSEA* root mean square error of approximation, Δ*RMSEA* change in RMSEA relative to the less constrained model, *Configural* unconstrained model, *Metric* factor loadings constrained equal across groups, *Scalar* factor loadings and item intercepts constrained equal across groups

Dashes indicate that no comparison was applicable

Single-group CFAs indicated good to excellent model fit across gender and age groups (CFI ≥ 0.996; RMSEA ≤ 0.088). Multigroup CFAs showed excellent fit for the configural models across both gender (χ² (2) = 5.83, CFI = 0.999, RMSEA = 0.024) and age (χ² (5) = 7.61, CFI = 1.000, RMSEA = 0.013), supporting comparable factor structures. Constraining factor loadings to equality across groups did not significantly worsen model fit for either gender (Δχ² = 1.22, *p* = .54; ΔCFI = 0.001; ΔRMSEA = 0.009) or age (Δχ² = 5.52, *p* = .70; ΔCFI < 0.001), indicating support for metric invariance.

Imposing additional equality constraints on item intercepts (scalar invariance) resulted in statistically significant χ² difference tests for both gender (Δχ² = 11.37, *p* = .010) and age (Δχ² = 32.19, *p* = .001). However, changes in approximate fit indices were small and within recommended thresholds (gender: ΔCFI = 0.002, ΔRMSEA = 0.008; age: ΔCFI = 0.003, ΔRMSEA = 0.014), providing approximate support for scalar invariance across both grouping variables. It should be noted that single-group models for each age band had only one degree of freedom, limiting the stability and interpretability of fit indices at this level. The elevated RMSEA observed for the 70–74 age group (RMSEA = 0.088) likely reflects this instability.

A two-factor multigroup CFA was estimated across men and women. Factor loadings were constrained equal across groups, and item intercepts were constrained equal for latent mean comparison. Model fit was acceptable, χ² (6) = 38.57, CFI = 0.995, RMSEA = 0.041. Relative to men, women did not differ significantly on factor depression (ΔM = 0.039, *p* = .066) but scored significantly higher on anxiety (ΔM = 0.107, *p* < .001).

### Convergent validity

To evaluate the convergent validity of the PHQ-4, bivariate Pearson correlations were calculated with conceptually related constructs across four survey waves. PHQ-4 scores were negatively correlated with quality of life in 2021 (*N* = 820, *r* = − .595), resilience in 2022 (*N* = 759, *r* = − .504), sense of coherence in 2023 (*N* = 814, *r* = − .733), and well-being in 2023–2024 (*N* = 798, *r* = − .679). All correlations were significant (*p* < .001) and in the expected direction, supporting the convergent validity of the measure.

### Normative values

Detailed normative percentile ranks for PHQ-4 scores by age and gender are provided in Tables 9 and 10. Detailed PHQ-2 and GAD-2 percentile ranks are provided in Appendix A [see Additional file 1, Tables A1 and A2].

**Table 9:** PHQ-4 norms for the total sample and men aged 60 years and older

Raw score	All participants	Men 60–64	Men 65–69	Men 70–74	Men 75–79	Men 80+
*N*	3208	453	376	341	165	209
M (SD)	2.0 (2.3)	1.6 (2.2)	1.7 (2.3)	1.7 (2.1)	1.9 (2.2)	2.5 (2.5)
0	38.8	47.9	44.9	41.1	42.4	30.6
1	53.7	63.6	61.2	57.8	56.4	43.5
2	67.7	74.8	72.1	76.2	69.1	57.4
3	77.7	83.2	80.9	84.5	75.2	67.9
4	88.2	91.4	89.4	90.9	86.7	81.3
5	91.8	93.2	91.8	94.1	92.1	86.6
6	94.5	95.4	94.1	96.2	94.5	90.9
7	96.4	96.9	96.3	97.1	97.6	94.3
8	98.0	98.5	97.6	97.7	100.0	98.1
9	98.9	98.9	98.9	98.8	100.0	99.0
10	99.5	99.3	99.2	99.7	100.0	100.0
11	99.7	99.8	99.7	99.7	100.0	100.0
12	100.0	100.0	100.0	100.0	100.0	100.0

Values for each raw score are cumulative percentile ranks (%), representing the percentage of participants in the reference group who scored at or below that value

*M* mean, *SD* standard deviation

**Table 10:** PHQ-4 norms for the total sample and women aged 60 years and older

Raw score	All participants	Women 60–64	Women 65–69	Women 70–74	Women 75–79	Women 80+
*N*	3208	459	416	327	222	238
M (SD)	2.0 (2.3)	2.0 (2.3)	1.7 (2.1)	2.2 (2.1)	2.3 (2.3)	2.8 (2.7)
0	38.8	38.3	43.3	28.1	32.4	26.9
1	53.7	53.4	57.5	46.5	45.5	36.6
2	67.7	68.0	70.7	62.4	59.5	53.4
3	77.7	78.9	81.7	75.2	68.5	66.4
4	88.2	88.2	90.1	88.7	85.6	81.9
5	91.8	91.3	94.7	92.4	92.8	84.9
6	94.5	94.3	96.2	96.3	95.0	88.2
7	96.4	96.5	97.4	96.9	97.3	91.6
8	98.0	98.0	98.3	98.5	98.6	95.0
9	98.9	98.7	99.5	99.1	98.6	97.1
10	99.5	99.1	100.0	100.0	99.1	98.7
11	99.7	99.6	100.0	100.0	99.5	98.7
12	100.0	100.0	100.0	100.0	100.0	100.0

Values for each raw score are cumulative percentile ranks (%), representing the percentage of participants in the reference group who scored at or below that value

*M* mean, *SD* standard deviation

## Discussion

The current findings contribute to the research of mental health in later life by providing normative data for the PHQ-4 for older adults aged 60 years and over. Percentile norms derived from the combination of four nationwide representative German samples allow meaningful comparison of individual scores with age- and gender-specific reference groups. These percentile ranks describe relative standing within the reference distribution and are not equivalent to the established PHQ-2 and GAD-2 screening cut-offs, which indicate whether further clinical evaluation may be warranted. Detailed normative values for older adults are especially significant, because of the specific aging-related changes that older adults face and the potentially severe consequences of poor mental health on their physical health and well-being [71].

The current study also expands our understanding of the psychometric properties of PHQ-4 in older adults. The internal consistency of PHQ-4 was 0.876 measured with both Cronbach’s alpha and McDonald’s omega, which are excellent values for a brief instrument and consistent with the literature regarding PHQ-4 [72, 73]. The subscales GAD-2 and PHQ-2 were also found to have good internal consistency (Cronbach’s alpha 0.795 and 0.832), despite consisting of only two items each. Item-level analyses indicated positively skewed but acceptable distributions, strong item–total correlations and no evidence that removal of any item would improve reliability, supporting the adequacy of all PHQ-4 items.

The results of the conducted CFA were in accordance with the literature and yielded a better fit of the two-factor model than the one-factor model [62]. The two-factor model, distinguishing between anxiety and depressive symptoms as measured by the GAD-2 and PHQ-2 items, demonstrated an excellent fit to the data [64]. This conclusion was supported by the ordinal WLSMV sensitivity analysis, which produced strong standardized loadings, good approximate fit, and a depression–anxiety correlation that was nearly identical to the maximum-likelihood estimate. The two-factor model also reached configural, metric and scalar invariance across both gender and age, indicating that the underlying factor structure and factor loadings are comparable between groups and that group differences can be meaningfully interpreted [65]. However, with only one remaining degree of freedom, this near-perfect fit is statistically expected for a model with so few estimated parameters and should not be interpreted as strong evidence of perfect model fit. The latent correlation between the depression and anxiety factors was high (*r* = .89), consistent with the substantial symptom overlap reported in older adults [14]. Although the two-factor model remained statistically preferable to the one-factor solution, this overlap indicates that depression and anxiety, while empirically separable, may not represent fully distinct constructs in this population.

PHQ-4 was compared to a wide variety of other questionnaires to evaluate its convergent validity. Overall, the correlation between PHQ-4 and the chosen instruments was in the expected theoretical direction and strength. Because each comparison instrument was administered in a different survey wave, the correlations support convergent validity in a broad sense rather than as evidence from one consistent set of validation measures. Our current findings provide further support for the psychometric properties of the PHQ-4. It can be employed as a solid screening questionnaire for mental health distress in older adults.

The distribution of PHQ-4 scores revealed slightly lower levels of psychological distress in the older adult population compared with the general population, with more than two thirds of participants falling within the normal range and approximately one third exhibiting at least mild symptom levels [52]. Based on established screening cut-offs, 11.2% of participants screened positive for probable depression and 8.6% for probable anxiety. Compared with the general population (probable anxiety 16.1%, probable depression 19.1%), adults aged ≥ 60 years in our sample showed lower positive-screening rates for anxiety (8.6%) and depression (11.2%) [52]. These figures represent screening classifications rather than diagnostic prevalence. Although the proportions were relatively small, they indicate that a substantial minority may warrant further assessment. Higher overall psychological distress was observed among women—primarily because of higher anxiety symptoms—and in the oldest age group.

To examine whether the COVID-19 pandemic substantially biased the results, psychological distress levels were compared across survey waves spanning the pandemic and post-pandemic periods. Although statistically significant differences were observed, effect sizes were small (η² ≈ 0.009–0.010), indicating that survey year explained only about 1% of the variance in symptoms. The temporal effects found were modest and symptom levels remained largely stable across assessment waves.

Our finding that women report more anxiety symptoms than men in later life is in line with previous research and with evidence that women tend to report higher levels of anxiety symptoms across the life span, a pattern that appears to persist into older age [74]. Widowhood and living alone are more common among women due to their longer life expectancy and may increase vulnerability to anxiety and depressive symptoms in later life [75]. In addition, cumulative socioeconomic disadvantage across the life course, including lower lifetime earnings, pension inequality, and financial strain, may also contribute to elevated psychological distress in later life [76, 77].

Psychological distress scores were higher in the older age groups. Evidence from population-based cohort studies of the oldest old suggests that this could be due to aging-related changes such as poor physical health, multimorbidity, frailty, disability, and cognitive impairment [78–80].

The number of older adults is expected to continue to increase. This means that even relatively modest prevalence rates of anxiety and depression translate into a substantial absolute number of affected individuals. High-quality screening is particularly valuable because it enables efficient identification of individuals most likely to benefit from intervention, thereby supporting more efficient targeting of clinical resources and treatment efforts. Evidence indicates that psychological treatment, particularly cognitive behavioral therapy (CBT), is effective for reducing symptom burden and improving clinical outcomes in older adults with anxiety and depression [81, 82]. Beyond symptom relief, effective treatment has been shown to improve physical and role functioning and to mitigate functional impairment, thereby reducing the risk of functional decline in later life [83, 84]. Moreover, untreated anxiety and depression in older adults are associated with increased health care utilization and poorer health-related quality of life, whereas collaborative and evidence-based treatment models are linked to improved outcomes and greater efficiency of care delivery [85, 86]. Beyond economic implications, improved mental health in older age is associated with enhanced quality of life and psychosocial functioning [39, 87, 88].

Several limitations of the present study should be considered when interpreting the current findings. First, the cross-sectional nature of the analyses limits interpretations about temporal stability and causal mechanisms. Future research should therefore examine test–retest reliability and longitudinal measurement invariance of the PHQ-4 in older populations. Second, while the sample included adults of very old age, more fine-grained normative values for the oldest-old will become increasingly important, as this group is both rapidly growing and, as our results suggest, especially vulnerable. Third, reliance on self-report screening measures rather than clinical diagnoses may have led to misclassification of mental health status [89]. A further limitation concerns the use of unweighted data. Although the four source surveys were designed to be representative of the German population, the composition of the pooled sample may still differ slightly from that of the population aged 60 years and older. The normative values and prevalence estimates may therefore be modestly affected by the under- or overrepresentation of certain sociodemographic groups. In addition, age-specific response rates were unavailable for adults aged 60 years and older. The magnitude and direction of potential nonresponse and selection bias in this age group could therefore not be determined. The participants may represent a positively selected group, as they were willing and able to participate and were living in private households rather than in nursing homes or hospitals. More vulnerable, frail, or institutionalized older adults may therefore be underrepresented. Their inclusion might have resulted in higher PHQ-4 scores and a more pronounced age-related increase in anxiety and depression [10]. The normative values should consequently be interpreted primarily as applying to older adults living in private households. Percentile estimates for the smallest normative subgroups (men aged 75–79 and 80+, women aged 80+) should also be interpreted with caution, as extreme percentile ranks are less stable when based on limited sample sizes. Finally, future studies could further strengthen external validity by comparing PHQ-4 scores with longer diagnostic instruments for depression and anxiety, cognitive functioning measures, and treatment status, thereby providing a more comprehensive picture of mental health in later life.

## Conclusion

The PHQ-4 is an economical, reliable and valid screening instrument for psychological distress among community-dwelling older adults in Germany. It is not a diagnostic instrument; elevated scores indicate the need for further clinical assessment rather than the presence of a mental disorder. Although most participants reported low levels of psychological distress, a minority of older adults screened positive for probable depression or anxiety. Women reported higher anxiety symptoms, while overall psychological distress was highest in the oldest age group. The age- and gender-specific normative values presented here may enhance score interpretation, but they primarily apply to older adults living in private households and should not be generalized without caution to institutionalized or hospitalized populations. Given its brevity and psychometric properties, the PHQ-4 may be useful for large-scale screening and for identifying individuals who may benefit from additional assessment.

## Supplementary Information


Additional file 1: Appendix A. Detailed PHQ-2 and GAD-2 normative tables.


## Data Availability

The dataset analysed during the current study is available in the Zenodo repository, https://doi.org/10.5281/zenodo.20138404

## Abbreviations

ANOVA: Analysis of variance
BIC: Bayesian information criterion
BRS: Brief Resilience Scale
CBT: Cognitive behavioral therapy
CFA: Confirmatory factor analysis
CFI: Comparative fit index
EFA: Exploratory factor analysis
EUROHIS-QOL: European Health Interview Survey-Quality of Life 8-item index
GAD-2: Generalized Anxiety Disorder-2
M: Mean
PHQ-2: Patient Health Questionnaire-2
PHQ-4: Patient Health Questionnaire-4
RMSEA: Root mean square error of approximation
SD: Standard deviation
SOC-L9: Sense of Coherence Scale–9
SPSS: Statistical Package for the Social Sciences
SRMR: Standardized root mean square residual
TLI: Tucker–Lewis index
WHO-5: World Health Organization-Five Well-Being Index
WLSMV: Weighted least squares mean- and variance-adjusted estimator
