# Supplementary material for: Psychometric properties and age- and gender-specific normative values of the Patient Health Questionnaire-4 among older adults aged 60 years and above
Source: BMC Geriatr. 2026 Aug 28;26:1109. doi: 10.1186/s12877-026-08172-5 (PMC13525647; doi:10.1186/s12877-026-08172-5)
Supplement: Supplementary file 1 [file 12877_2026_8172_MOESM1_ESM.docx]

**Additional file 1**

*Psychometric properties and age- and gender-specific normative values of the Patient Health Questionnaire-4 among older adults aged 60 years and above*

## Appendix A. Detailed PHQ-2 and GAD-2 normative tables

The following tables provide age- and gender-specific cumulative percentile ranks for the PHQ-2 and GAD-2.

**Table A1**

*Normative values of the PHQ-2 in adults aged 60 years and older*

*Panel A. Total sample and men*

| **Raw score** | **Total sample** | **Men**  **60–64** | **Men**  **65–69** | **Men**  **70–74** | **Men**  **75–79** | **Men**  **80+** |
| --- | --- | --- | --- | --- | --- | --- |
| N | 3208 | 453 | 376 | 341 | 165 | 209 |
| M (SD) | 1.1 (1.3) | 0.9 (1.2) | 1.0 (1.3) | 1.0 (1.3) | 1.1 (1.3) | 1.5 (1.4) |
| 0 | 45.9 | 53.6 | 51.6 | 46.6 | 46.7 | 34.9 |
| 1 | 66.3 | 73.1 | 71.0 | 70.4 | 61.8 | 53.6 |
| 2 | 88.8 | 91.2 | 88.8 | 90.6 | 87.3 | 79.9 |
| 3 | 93.4 | 94.9 | 92.6 | 93.8 | 92.7 | 88.5 |
| 4 | 97.8 | 97.8 | 97.3 | 97.7 | 98.8 | 97.6 |
| 5 | 99.2 | 99.3 | 99.5 | 99.1 | 99.4 | 99.0 |
| 6 | 100.0 | 100.0 | 100.0 | 100.0 | 100.0 | 100.0 |

*Panel B. Total sample and women*

| **Raw score** | **Total sample** | **Women**  **60–64** | **Women**  **65–69** | **Women**  **70–74** | **Women**  **75–79** | **Women**  **80+** |
| --- | --- | --- | --- | --- | --- | --- |
| N | 3208 | 459 | 416 | 327 | 222 | 238 |
| M (SD) | 1.1 (1.3) | 1.0 (1.3) | 0.9 (1.2) | 1.2 (1.2) | 1.2 (1.3) | 1.6 (1.5) |
| 0 | 45.9 | 48.1 | 53.1 | 37.3 | 39.2 | 31.5 |
| 1 | 66.3 | 68.2 | 74.8 | 63.9 | 57.2 | 48.3 |
| 2 | 88.8 | 90.0 | 91.8 | 89.6 | 88.3 | 82.8 |
| 3 | 93.4 | 94.3 | 95.4 | 95.1 | 94.6 | 87.0 |
| 4 | 97.8 | 98.3 | 98.8 | 98.5 | 98.2 | 93.7 |
| 5 | 99.2 | 99.1 | 99.5 | 99.4 | 99.1 | 97.9 |
| 6 | 100.0 | 100.0 | 100.0 | 100.0 | 100.0 | 100.0 |

*Note.* Values for each raw score are cumulative percentile ranks (%), representing the percentage of participants in the reference group who scored at or below that value. M = mean; SD = standard deviation. The all-participants column is identical in both panels and is repeated to allow direct comparison with the age- and gender-specific groups.

**Table A2**

*Normative values of the GAD-2 in adults aged 60 years and older*

*Panel A. Total sample and men*

| **Raw score** | **Total sample** | **Men**  **60–64** | **Men**  **65–69** | **Men**  **70–74** | **Men**  **75–79** | **Men**  **80+** |
| --- | --- | --- | --- | --- | --- | --- |
| N | 3208 | 453 | 376 | 341 | 165 | 209 |
| M (SD) | 0.9 (1.2) | 0.7 (1.1) | 0.7 (1.2) | 0.6 (1.1) | 0.7 (1.1) | 1.0 (1.2) |
| 0 | 54.4 | 63.4 | 61.7 | 63.6 | 61.8 | 47.4 |
| 1 | 73.8 | 81.7 | 76.9 | 81.8 | 77.6 | 67.9 |
| 2 | 91.4 | 92.9 | 92.8 | 94.1 | 91.5 | 86.6 |
| 3 | 95.8 | 96.2 | 96.0 | 97.7 | 97.0 | 94.7 |
| 4 | 98.6 | 99.1 | 98.4 | 98.8 | 99.4 | 99.5 |
| 5 | 99.5 | 99.6 | 99.5 | 99.4 | 100.0 | 100.0 |
| 6 | 100.0 | 100.0 | 100.0 | 100.0 | 100.0 | 100.0 |

*Panel B. Total sample and women*

| **Raw score** | **Total sample** | **Women**  **60–64** | **Women**  **65–69** | **Women**  **70–74** | **Women**  **75–79** | **Women**  **80+** |
| --- | --- | --- | --- | --- | --- | --- |
| N | 3208 | 459 | 416 | 327 | 222 | 238 |
| M (SD) | 0.9 (1.2) | 0.9 (1.2) | 0.8 (1.1) | 1.0 (1.1) | 1.0 (1.2) | 1.2 (1.4) |
| 0 | 54.4 | 51.4 | 52.9 | 45.3 | 45.9 | 42.9 |
| 1 | 73.8 | 72.1 | 76.0 | 67.0 | 65.8 | 62.2 |
| 2 | 91.4 | 91.1 | 92.3 | 92.4 | 89.6 | 85.7 |
| 3 | 95.8 | 94.3 | 96.6 | 96.3 | 96.4 | 92.4 |
| 4 | 98.6 | 98.0 | 98.3 | 99.7 | 98.6 | 96.6 |
| 5 | 99.5 | 99.3 | 99.8 | 99.7 | 99.5 | 98.3 |
| 6 | 100.0 | 100.0 | 100.0 | 100.0 | 100.0 | 100.0 |

*Note.* Values for each raw score are cumulative percentile ranks (%), representing the percentage of participants in the reference group who scored at or below that value. M = mean; SD = standard deviation. The all-participants column is identical in both panels and is repeated to allow direct comparison with the age- and gender-specific groups.
